# Medical History from the Earliest Times

**Published:** 1892-12-31

**Authors:** E. T. Withington


### Medical History from the Earliest Times.

By E. T. Withington, M.A., M.B. Oxon.

### XXXVII.—THE LADIES OF SALERNO.

The above is the more courteous translation of *Mulieries Salernitanæ* a phrase of very frequent occurrence in the writings of the Salernitan masters, and usually introducing an account of some form of treatment employed either by the women of Salerno generally, or by particular ladies connected with the medical school, some of whom are mentioned by name. Magister Bernardus is especially fond of referring to "the ladies of Salerno," and it may be of one of them that he quotes, apropos of the virtues of the load-stone the pretty line *Ut ferrum magnes, juvenes sic attrahi Agnes.* They were sometimes not above practical jokes. "The ladies of Salerno," says Bernard, "sprinkle roses with powdered euphorbium and give them to young men to smell, whereupon they instantly begin to sneeze." Here are some of his other references. "The Salernitan ladies, when they have long mourned for their dead friends, take a young pig's heart, stuff it with spices, and bake it in paste, then they eat it and at once forget their sorrow; but men do not eat animals' hearts either because they cause forgetfulness, or because they are indigestible." "The *mulieres Salernitanæ* mix the root of spatula fœtida (fetid iris) with honey, and anoint their faces therewith to remove wrinkles." "They use aloes and rosewater for swellings on the face, but absinth is better than aloes." Magister Maurus observes, "The Salernitan ladies treat dropsy as follows: they go to the woods and collect plants indiscriminately, diurectic and others, maidenhair, hepatica, &c., &c. These they boil in salt water, and the patient first inhales the steam, then drinks, and finally bathes in it. While bathing they give him an electuary, such as—(here follows a long list)—or all these mixed together, and the juice of chick-pea (cicer). They repeat this five times, and they have cured many." Among other notices we may mention the following: "The Salernitan ladies steep bryony root in honey and anoint their faces with it, which gives them a marvellous blush." "The ladies of Salerno give children poppy seeds in milk." "They make cakes of pellitory (parietaria), flour, and water, which are good for indigestion." "A certain lady of Salerno has proved that cyclamen juice is good for hæmorrhoids:" "For dryness and roughness of the hair, mix bole armeniac with hot water, and after washing the head pour this on it, then wash again with warm water; *sic operant mulieres Salernitanæ*." The other references deal chiefly with diseases of women, and all are medical, but in later times the Salernitan ladies seem to have adopted more general practice, and we find Bruno of Calabria closing his lament on the sad state of surgery in his days (13th century) with the exclamation, "Not only amateurs (idiotæ) and laymen, but what is yet more horrible and indecent, vile and presumptuous females have now usurped and abuse our art."

When Rudolph the monk went to Salerno in 1059, he found, we are told, no one who could meet him in argument save "a certain learned matron" who is believed to have been Trota, or Trotula, the most famous of all the Salernitan ladies and the supposed authoress of a still existing treatise, "On Diseases of Women." The authenticity of this work is somewhat doubtful, but Trotula is frequently quoted by other Salernitans, and the following extracts show that she did not confine herself to gynæcology: "For toothache rub up rue and pepper, and put it in the tooth for one night, but if this is no good cauterize the tooth with a thin iron wire through a funnel, or put a little sugar in it." "For colic due to chill, or damp, or crudity of the humours, give hot water internally, and apply a sponge or cloth dipped in hot wine and slightly squeezed out externally." The treatise, "De Morbis Mulierum," contains two excellent chapters on the management of the new-born baby and the choice of a nurse, which are, however, too long to quote, and ends with an important section on cosmetics. Here is a simple recipe. "To make the hair golden, take of elder bark, flowers of broom, yolk of egg, and saffron equal parts; boil them in water; skim off what floats on the surface and use as pomade." Trotula's fame in medicine extended beyond Italy, and lasted till the thirteenth century. The French poet, Ruteboeuf, has preserved or imitated an oration by a travelling quack of that period. He is, he declares, no ordinary charlatan, but one of the special agents sent out by the famous Madame Trote of Salerno to catch strange beasts from which she manufactures her world-renowned ointments. Trotula is usually entitled the "Magistra" or "Maestra;" the degree of "Doctor" was introduced later and was attained by another Salernitan lady, Constanza Calenda, A.D. 1430. Besides these we hear of Abella, Rebecca Guarna, and Mercuriada, the last of whom wrote a treatise on surgery, which perhaps excited the wrath of Bruno of Calabria.

Some of the Salernitan adies have become famous in general history. Sichelgaita the Duchess, whose amazonian feats at the side of her husband, Robert Guiscard, are known to readers of Scott and Gibbon, studied medicine at Salerno, her native town, and paid special attention to toxicology. She was naturally anxious that her own son, Roger, should obtain the birthright of his half-brother, Bohemund, and hearing that that famous crusader had gone to Salerno to recruit his health, she sent his medical attendant, one of her old teachers, a box containing a slow but effectual poison. The physician took the hint and administered the drug; but Duke Robert somehow became suspicious of what was going on, and calling for his Bible and his sword, he swore on the former that he would plunge the latter into his wife's heart on the day he heard of his son's death. Sichelgaita was equal to the occasion; she at once sent a trusty messenger to Salerno with a never-failing antidote, and "by the blessing of God, who had ordained Bohemund to be a scourge of the infidel," he recovered, but had for ever afterwards a remarkably pallid countenance. Some years later Sichelgaita succeeding in poisoning her husband and in making her son Roger his successor. For the credit of the duchess and the doctors it should be added that this story rests only on the evidence of a monk, Ordericus Vitalis, who wrote 50 years after the supposed events, and that its latter part is contradicted by other historians, who assert that Sichelgaita died before her husband.

The following is yet more tragic, and somewhat better authenticated. Stephania, who lived at the close of the tenth century, was very skilful in "Galenic matters," and though we are not told that she studied at Salerno, she can hardly have obtained such knowledge anywhere else. Her beauty was equal to her learning, and she married in early youth Crescentius, a noble Roman, who had conceived the ambition of reviving the ancient glories of the Republic. Unfortunately, the Emperor, Otto III., resolved at the same time to make the eternal city the centre of the Holy Roman Empire, and Crescentius, deserted by the fickle populace, was beseiged by overwhelming forces. In defiance of the terms of capitulation (if we may trust Italian writers) the head of the consul and patrician was stuck upon the walls of St. Angelo, and his beautiful wife submitted to the insults of the German soldiery. Her subsequent history has been compared to a Greek tragedy. "She ensnared the young Emperor by her beauty, and slew him with a lingering poison." The chronicler, Landulph, tells the story at length. Otto lay sick at Paterno, and Stephania treated him for twelve days "with Hippocratic and Galenic remedies" so successfully that the ordinary attendants were dismissed, and only she and her maid allowed access to the patient. Then she proclaimed that he must be wrapped naked in the skin of a freshly-killed deer, a well-recognised mode of treatment, the vigour and supposed longevity of the animal being believed to pass over into the patient. The skin was procured, but Stephania smeared it with baleful and corrosive poisons, and, wrapped in this second Nessus shirt, the Emperor perished in agonies, in the twenty-second year of his age, January 23rd, 1002. The story is repeated, with variations, by several reliable writers, but it is hard to believe that Stephania not only escaped punishment, but proceeded to attack the Emperor's friend, Pope Gerbert. (Sylvester II) Neither his medicine nor his magic could save the supreme Pontiff from the vengeance of an injured woman, and he died, paralysed and speechless, within sixteen months of the Emperor; but what may have been an attack of apoplectic aphasia was variously ascribed by mediæval writers to the poisons of Stephania or to the more direct action of that evil spirit to whom, in exchange for Moslem wisdom, the young student of Cordova had, years before, bartered his soul.

The school of Salerno is probably most widely known through Longfellow's version of the "Golden Legend," the original of which dates from the twelfth century, and the Salernitan ladies are mentioned in another romance of the same period. Marie de France relates in one of her "Lais" how a lover, before he could obtain his mistress, was required to carry her in his arms to the top of a hill. In vain does the lady starve herself to the uttermost; her weight is still such as would render the feat a dangerous one. Then she bethinks her of an aunt, who has studied the healing art for thirty years at Salerno, and who, therefore, has no difficulty in composing a strengthening medicine of marvellous potency. But the hero, unfortunately, refuses to use such aids, and the story ends tragically

